# Distributed Tensor Decomposition for Large Scale Health Analytics

**DOI:** 10.1145/3308558.3313548

**Published:** 2019-05

**Authors:** Huan He, Jette Henderson, Joyce C. Ho

**Affiliations:** Emory University, Atlanta, Georgia; CognitiveScale, Austin, Texas; Emory University, Atlanta, Georgia

**Keywords:** Web Mining, User-Generated Content, Health Analytics, Tensor Decomposition, Distributed Algorithm, Apache Spark

## Abstract

In the past few decades, there has been rapid growth in quantity and variety of healthcare data. These large sets of data are usually high dimensional (e.g. patients, their diagnoses, and medications to treat their diagnoses) and cannot be adequately represented as matrices. Thus, many existing algorithms can not analyze them. To accommodate these high dimensional data, tensor factorization, which can be viewed as a higher-order extension of methods like PCA, has attracted much attention and emerged as a promising solution. However, tensor factorization is a computationally expensive task, and existing methods developed to factor large tensors are not flexible enough for real-world situations.

To address this scaling problem more efficiently, we introduce SGranite, a distributed, scalable, and sparse tensor factorization method fit through stochastic gradient descent. SGranite offers three contributions: (1) Scalability: it employs a block partitioning and parallel processing design and thus scales to large tensors, (2) Accuracy: we show that our method can achieve results faster without sacrificing the quality of the tensor decomposition, and (3) FlexibleConstraints: we show our approach can encompass various kinds of constraints including l2 norm, l1 norm, and logistic regularization. We demonstrate SGranite’s capabilities in two real-world use cases. In the first, we use Google searches for flu-like symptoms to characterize and predict influenza patterns. In the second, we use SGranite to extract clinically interesting sets (i.e., phenotypes) of patients from electronic health records. Through these case studies, we show SGranite has the potential to be used to rapidly characterize, predict, and manage a large multimodal datasets, thereby promising a novel, data-driven solution that can benefit very large segments of the population.

## 1. INTRODUCTION

Increasingly large amounts of health-related data are released on the Internet and have great potential for enabling better disease surveillance and disease management. As a motivating example, search activities on diseases such as influenza can be used and correlated with actual influenza surveillance data. Estimation of influenza-like illness (ILI) rates is a well-studied task [25, 32], Google Flu Trends, while flawed, demonstrated a link between influenza related search queries and the Centers for Disease Control and Prevention’s (CDC) ILI rates[19]. Similarly, programs such as the National Institute of Health’s All for Us NIH [2], are looking to gather data and make it publicly available to researchers to enable precision medicine. Extracting influenza patterns or clinical characteristics from such high-dimensional data can pose challenges, even before considering whether the data has been appropriately labeled.

A vast majority of the algorithms for disease surveillance or disease prediction adopt a supervised learning approach, but the need for labels can limit the possible scope of the task. However, unsupervised learning methods such as tensor factorization have been successfully applied in many application domains including social network analysis [29, 30, 39] and health analytics [15, 16, 18, 22, 36]. Tensors can succinctly represent high-dimensional data, including various representations of time or different sources of data. For example, an existing work showed that factorizing a tensor that grouped ILI historical statistics by year, week, and region could tease out patterns that are commonly based on the weeks that influenza is highest, deliver insight into the degree to which regions are similar or different from one another in terms of influenza, and capture the changes in ILI intensity from one year to the next [13]. Moreover, a variety of constraints can be placed on the learned latent factors to extract meaningful patterns and reduce overfitting. Yet, efficient tensor decomposition of large datasets in the presence of such constraints can be challenging.

In this paper, we propose SGranite, a distributed tensor decomposition framework that can incorporate a variety of regularization terms to constrain the latent factors. In particular, we show that integrating three forms of regularization terms can achieve easier-to-interpret factors, provide robustness in the presence of noise, and map to existing domain knowledge. Moreover, SGranite is very fast and scalable. Using a Spark-based implementation, we demonstrate the ability to decrease computation time by distributing both the data as well as the parameters without sacrificing accuracy. To promote reproducibility, our code is open-sourced and available on GitHub^1^.

The contributions of our work can be summarized as follows:

- **Flexibility:** Our framework supports a variety of meaningful constraints such as sparsity, diversity, and distinguishability.
- **Scalability:** Our scalability analysis of SGranite on a large tensor constructed from healthcare data achieves near linearity speed-up as we scale to the number of machines. Moreover, our framework achieves at least a 4× speed-up compared to an existing state-of-the-art distributed tensor factorization method.
- **Accuracy:** Our empirical results in two health-related case studies show that incorporating the variety of constraints improves interpretability and robustness compared to the standard decomposition models.

Table 1 summarizes the contributions in the context of existing works.

## 2 BACKGROUND AND NOTATION

This section briefly introduces tensors and a popular tensor decomposition model. We refer the reader to [24, 34] for comprehensive overviews of practical tensor decompositions.

### 2.1 Tensors

Tensors are generalizations of matrices and vectors to higher dimensions. An N-way tensor is denoted as $X\in {\mathbb{R}}^{I_{1}\times I_{2}\times \cdots \times I_{N}}$ and each cell of the tensor represents the interactions between *N* types of data. Each dimension of the tensor is referred to as a mode. Tensors can be unfolded or flattened as a matrix, which is called *matricization*. X_(*n*)_ denotes the matricization of tensor X along mode-*n*. Table 2 lists the operations and symbols used in this paper.

*Definition 2.1.* A rank-one N-way tensor is the outer product of *N* vector

$$X=a^{\left(1\right)}\circ a^{\left(2\right)}\circ \cdots \circ a^{\left(N\right)}.$$

Each element of a rank-one tensor is the product of the corresponding vector elements (i.e.,$x_{i_{1}i_{2}i_{3}\cdots i_{n}}=a_{i_{1}}^{\left(1\right)}\circ a_{i_{2}}^{\left(2\right)}\circ \cdots \circ a_{i_{N}}^{\left(N\right)}$).

### 2.2 Tensor Decompositions

**Figure 1:**
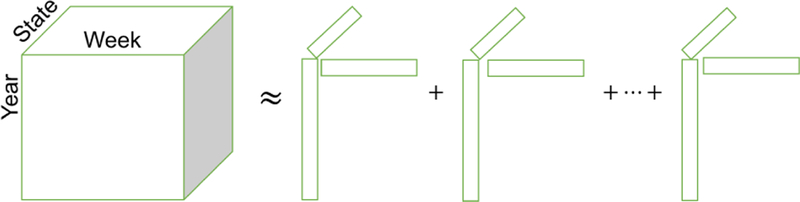
An example of CP decomposition for influenza search data. A tensor is constructed of time series data is decomposed into the weighted sum of rank-one tensors based on the minimization of an objective function. Each rank-one tensor, formed by taking the outer product of factor vectors, constitutes a latent factor.

The CANDECOMP / PARAFAC (CP) model [7, 14] is one of the most popular and well-studied tensor decomposition method. In CP decomposition, the tensor is factored into a sum of rank-one tensors:

$$X\approx M=\sum_{r=1}^{R}{\text{A}}^{\left(1\right)}\left(:,r\right)\circ {\text{A}}^{\left(2\right)}\left(:,r\right)\circ \cdots \circ {\text{A}}^{\left(n\right)}\left(:,r\right),$$

where **A**^(*n*)^(:, *r* ) is the r th column of **A**^(*n*)^. CP-decomposition can be also expressed as $\left[\left[\lambda ;{\text{A}}^{\left(1\right)};{\text{A}}^{\left(2\right)};\cdots ;{\text{A}}^{\left(n\right)}\right]\right]$, where λ is a vector of the weights $\lambda_{r}$. Several benefits of the CP decomposition includes its intuitive output structure, uniqueness property that makes the model reliable to interpret, and the ability to learn a model even with relatively small amount of observations [24]. Figure 1 provides an example of the CP decomposition for an influenza-based tensor, where each rank-one tensor represents a pattern over time for a group of states and a set of search queries.

The CP decomposition, ${\text{A}}^{\left(1\right)},{\text{A}}^{\left(2\right)},\cdots ,{\text{A}}^{\left(n\right)}$, is performed by minimizing the loss between X and M as defined by an objective function. The form of the objective function is determined by assumptions about how the data in the tensor was generated. A standard method of fitting a CP decomposition is the least squares formulation, which assumes the random variation in the tensor data follows a Gaussian distribution. Unfortunately, this may not be appropriate for count data, which is common in many applications including those considered in this paper [17].

A more appropriate objective function for count data assumes the underlying distribution of the data is Poisson [8]. This assumption results in an objective function that minimizes the Kullback-Leibler (KL) divergence:

$$f\left(M\right)=\sum_{i}m_{i}-x_{i}\text{log}m_{i}.$$

There have been several recent works to accelerate the computational speed of the CP decomposition. FlexiFact partitions the tensor into smaller tensors that are then decomposed in parallel using Apache Hadoop. While their method scales well with the input size, the model is not well-suited for count data and performs excessive disk operations. More recently, DisTenC, a Spark-based distributed tensor completion algorithm was proposed that regularizes the trace norm of the tensor [11]. Similar to FlexiFact, it is designed for numeric data and does not support any regularization or constraints on the factors.

## 3 SGRANITE

We propose SGranite, a distributed and flexible constrained CP model, to impose a variety of constraints on the latent factors. Our algorithm uses distributed stochastic gradient descent (DSGD) approaches to scale the CP decomposition on count data to huge datasets. SGranite has the following benefits:

- Simultaneously supports multiple constraints on the factor matrices.
- Learns patterns even when data cannot be stored on a single server.
- Maintains computational efficiency across a large number of workers.

A distributed framework for incorporating a variety of constraints in CP decomposition is appealing for several reasons including the ability to extract patterns from large datasets that cannot be readily stored on a centralized server, to encode prior knowledge, to improve interpretability, and to democratize high-dimensional learning by running on standard commodity servers.

In this section, we will first provide a general overview and then formulate the optimization problem.

### 3.1 General Optimization Problem

SGranite, builds on several existing nonnegative CP decomposition algorithms to model sparse count data using the Poisson distribution [8, 16]. Let X denote an observed tensor constructed from count data with size $I_{1}\times I_{2}\times \cdots \times I_{N}$ and M represent a same-sized tensor of Poisson parameters for X. In addition to KL divergence, we introduce generalized constraints on the factor matrices, $R({\text{A}}^{\left(n\right)})$ to the objective function. Thus, the optimization problem is defined as:

(1) $$\text{min}f(M)=\sum_{\vec{i}}\left(m_{\vec{i}}-x_{i}\text{log}m_{\vec{i}}\right)+\sum_{k}\underset{\text{regularization terms}}{\underset{︸}{\beta_{k}R_{k}\left({\text{A}}^{\left(n\right)}\right)}}\;\;\;\text{s.t.}M=〚\lambda ;{\text{A}}^{\left(1\right)},\cdots ,{\text{A}}^{\left(N\right)}〛\;\;\;\;\;\lambda_{r}\geq 0,{\left\|{\text{a}}_{r}^{\left(n\right)}\right\|}_{1}=1,\;\forall r\;\;\;\;\;{\text{A}}^{\left(n\right)}\in {\left[0,1\right]}^{I_{n}\times R},\;\forall n$$

The Poisson parameters, m, can be determined by minimizing the negative log-likelihood of the observed data x. We also maintain the stochasticity (i.e., elements sum to 1) and non-negativity constraints (i.e., factor elements and weights, or λ, must be non-negative) that were introduced in the original CP-APR model [8].

### 3.2 Example of Useful Regularization Terms

Equation 1 supports a variety of regularization items,$R({\text{A}}^{\left(n\right)})$. While we describe three forms of special regularizations that are useful for analyzing health data, SGranite was developed to handle any regularization that is either smooth and differentiable or has an easy-to-compute proximal operator [31].

#### 3.2.1 Diversity on $A^{\left(n\right)}$.

For analyzing flu patterns or clinical characteristics of patient subgroups, it is preferable for the rank-one factor components to be distinct from each other. This allows domain experts to more easily interpret the patterns. While several mechanisms for encouraging diversity have been proposed [16, 21, 36], we adopt the angular penalty term in [16] that encourages diversity between rank-one tensors by penalizing overlapping elements. There are two benefits to this regularization. It does not require prior knowledge to construct a similarity matrix that is used in [21]. Similarly, it does not require the discovered patterns to be orthogonal to one another [36], which may be too restrictive. Under angular regularization, any element that has large values in multiple columns in the factor matrix are penalized. Thus, the angular penalty for the *n*^th^ factor matrix, **A**^(*n*)^, is formulated as follows:

$$R_{k}\left({\text{A}}^{\left(n\right)}\right)=\sum_{r=1}^{R}\sum_{p=1}^{r}\text{max}{\left(0,\frac{{\left(a_{p}^{n}\right)}^{T}a_{r}^{n}}{{\left\|a_{p}^{n}\right\|}_{2}{\left\|a_{r}^{n}\right\|}_{2}}-\theta_{n}\right)}^{2}$$

#### 3.2.2 Sparsity and Smoothness on $A^{\left(n\right)}$.

Sparsity and smoothness constraints have been introduced in a wide range of applications to improve interpretability and increase robustness to noise. Our framework supports a general class of *ℓ*_p_ penalties including simplex constraint term $\left({\left\|{\text{a}}_{r}\right\|}_{1}=1,a_{ir}\in \left[0,1\right]\right)$; *ℓ*_2_ regularization on the weight and the first factor matrix, $\lambda A^{\left(1\right)}$ to mitigate overfitting to large count data; and the *ℓ*_0_-norm regularization which caps the number of non-zeros elements in the factor.

We first consider the simplex constraint term, which can yield sparse factors while providing a probabilistic interpretation. For the *n*^th^ factor matrix, **A**^(*n*)^, we restrict the elements to lie on the *ℓ*_1_-ball of diameter s, where s is a user-specified parameter, such that:

$$R_{k}\left({\text{A}}^{\left(n\right)}\right)=\sum_{r=1}^{R}\left(s-{\left\|a_{r}^{\left(n\right)}\right\|}_{1}\right)$$

When *s* = 1, this results in the projection of the factor onto the probabilistic (or canonical) simplex [9]. By decreasing s to be less than 1, the resulting factors will be sparser.

The *ℓ*_2_-norm regularization was introduced in [16] to encourage terms in the factor matrix vectors to be similarsized. Together with the simplex projection, the interaction of these two regularizations achieved further sparsity by driving specific elements to 0 more quickly in a similar manner to the elastic net regularization [41].

$$R_{k}\left({\text{A}}^{\left(n\right)}\right)=\sum_{r=1}^{R}{\left\|a_{r}^{\left(n\right)}\right\|}_{2}$$

The *ℓ*_0_-norm regularization, introduced in [4], is an alternative to the simplex projection that limits the number of non-zero elements. While its usage in Equation 1 results in a non-convex optimization problem, the hard thresholding properties can yield easy to interpret factors (top-k elements). To perform hard-thresholding on the *n*^th^ factor matrix, **A**^(*n*)^, the regularization term is:

$$R_{k}\left({\text{A}}^{\left(n\right)}\right)=\sum_{r=1}^{R}{\left\|a_{r}^{\left(n\right)}\right\|}_{0}$$

#### 3.2.3 Discriminative Factors.

In some scenarios, the discovered patterns should be discriminative of a certain outcome of interest. For example, we may want to use the clinical characteristics to predict things like mortality or whether or not the patient is likely to be readmitted in 30 days. [21] introduced a logistic regression regularization that encouraged the derivation of latent factors that can distinguish in-hospital mortality outcomes. SGranite also adopts the regularization term to derive discriminative latent factors when such information exists. Without loss of generality, we assume that the first mode has labeled records. Then the discriminative regularization is of the form:

$$R_{k}\left({\text{A}}^{\left(1\right)}\right)=\text{log}P\left(A^{\left(1\right)},y\left|\theta \right.\right)$$

The probability of a sample a(i , :) (*i*^th^ row in A^(1)^) having the out-come of interest, $P\left(A^{\left(1\right)},y\left|\theta \right.\right)$, is obtained by training a logistic regression model on the factor matrix A^(1)^.

#### 3.2.4 Sparse, Diverse, and Discriminative Patterns.

To demonstrate the flexibility of SGranite, we introduce all three forms of regularization into our final optimization problem. Thus, the final objective function is:

(2) $$f\left(M\right)=\sum_{\vec{i}}\left(m_{\vec{i}}-x_{i}\text{log}m_{\vec{i}}\right)+\;\;\;\;\;\beta_{1}\sum_{n=1}^{N}\sum_{r=1}^{R}\sum_{p=1}^{r}\text{max}{\left(0,\frac{{\left(a_{p}^{n}\right)}^{T}a_{r}^{n}}{∥a_{p}^{n}{∥}_{2}∥a_{r}^{n}{∥}_{2}}-\theta_{n}\right)}^{2}+\;\;\;\;\;\beta_{2}\sum_{n=1}^{N}\sum_{r=1}^{R}\left(s-∥{\text{a}}_{r}^{\left(n\right)}{∥}_{2})+\beta_{3}\text{log}P(A^{\left(1\right)},y\left|\theta \right.\right)$$

### 3.3 SGD Updates

This section provides details of how to solve our optimization problem efficiently (Equation 2). SGranite uses an alternating minimization approach, cycling through each mode while fixing all the other modes. For each mode, the resulting subproblem is solved using stochastic gradient descent (SGD). To derive the SGD updates, we first rewrite the objective function as a scalar-valued function of the parameter vector y using the same approach as [3]. The pa-rameter vector y represents the vectorization of the factor matrices, with the weights λ absorbed into the first factor matrix.

$$y=\left[\begin{array}{c} vec(\lambda {\text{A}}^{\left(1\right)})\\ vec({\text{A}}^{\left(2\right)})\\ \vdots \\ vec({\text{A}}^{\left(n\right)}) \end{array}\right]$$

As a result, the gradients of the objective function can be formed by vectorizing the partial derivatives with respect to each component of this parameter vector:

$$\nabla f\left(y\right)=\left[vec(\frac{\partial f}{\partial {\text{A}}^{\left(1\right)}})\cdots vec(\frac{\partial f}{\partial {\text{A}}^{\left(n\right)}})\right]$$

For notational convenience, we also represent the matricized form of the tensor decomposition as:

$${〚\lambda ;{\text{A}}^{\left(1\right)},\cdots ,{\text{A}}^{\left(N\right)}〛}_{\left(n\right)}=\lambda {\text{A}}^{\left(n\right)}{\left({\text{A}}^{\left(-n\right)}\right)}^{T}$$

where

$${\text{A}}^{\left(-n\right)}={\text{A}}^{\left(N\right)}⊙\cdots ⊙{\text{A}}^{\left(n+1\right)}⊙{\text{A}}^{\left(n-1\right)}⊙\cdots ⊙{\text{A}}^{\left(1\right)}.$$

Thus, the partial derivatives of Equation 2 with respect to the factor matrix, **A**^(*n*)^ are the following:

(3) $$\frac{\partial f}{\partial A_{r}^{\left(n\right)}}=[1-X_{\left(n\right)}⊘Z_{\left(n\right)}]a_{r}^{\left(-n\right)}+\;\beta_{1}\sum_{p\neq r}\text{max}(0,g(a_{r}^{\left(n\right)},a_{p}^{\left(n\right)}))\frac{\partial g(a_{r}^{\left(n\right)},a_{p}^{\left.\left(n\right)\right)}}{\partial {\text{a}}_{r}^{\left(n\right)}}+\;\beta_{2}{\text{a}}_{r}^{\left(n\right)}+\beta_{3}y\frac{1}{1+\text{exp}({\text{A}}_{r}^{\left(1\right)})}\theta$$

We refer the reader to [16, 21] for the detailed derivation of the gradients.

For large datasets, the calculation of the derivatives simultaneously for all modes is computationally expensive. Thus, SGranite uses an SGD approach to avoid storing the entire tensor in memory. For faster convergence, we adopt a variant of SGD named Adaptive Moment Estimation (Adam) to adaptively update the learning rate [23]. Our preliminary experiments on a single machine showed that SGD with Adam converged faster and more accurately than using a fixed learning rate.

### 3.4 Parallel Algorithm using Spark

Although SGD scales well to sparse data, we would like to distribute the computation to achieve results faster. FlexiFact proposed distributing the computation by dividing the tensor such that no two blocks share any elements (along with any dimension) [6]. Thus, the SGD algorithm can be run in parallel on each block without sacrificing accuracy. We refer the reader to [6, 12] for detailed proof of convergence. SGranite uses the same approach to distribute the non-zero elements of the count tensor using this tensor partition. However, we note several main differences between our framework and Flexifact: (1) support for sparse, count data by using an appropriate objective function (KL divergence), (2) flexibility to incorporate a variety of constraints beyond sparsity and non-negativity, and (3) distributed computation using Apache Spark.

$$\begin{array}{l} \bar{\underline{\text{Algorithm}\;1\;\text{SGD updating process}\;\;\;\;\;\;\;\;\;\;\;}}\\ \;1:\;\text{for}\;l=1:L\;\text{do}\\ \;\;\;2:\;\;\;\;\text{Randomly select}\;n\;\text{samples}\\ \;\;\;3:\;\;\;\;\text{Calculate the gradients for samples using Equation}3\\ \begin{array}{l} \;\;\;4:\;\;\;\;\text{Compute the decaying averages of past and past squared}\\ \;\;\;\;\;\;\;\;\text{ gradients} \end{array}\\ \;\;\;5:\;\;\;\;\text{Take a step using averaged gradients}\\ \underline{\;\;\;6:\;\text{end for}\;\;\;\;\;\;\;\;\;\;\;\;\;\;\;\;\;\;\;\;} \end{array}$$

Unlike SGranite, FlexiFact uses the Hadoop Map-Reduce platform to distribute the data collection across multiple nodes. Unfortunately, a Hadoop workflow spends an exorbitant amount of time on disk operations, as it needs to read and write intermediary results on the disk. On the other hand, Apache Spark [40] has been proposed as an alternative that eliminates unnecessary disk operations for iterative algorithms. By performing the data analytic operations inmemory and in near real-time, Spark can achieve lower computation times. Thus, SGranite is developed using Spark to distribute the computation.

#### 3.4.1 Tensor Partition.

First, we define a stratum as a set of independent blocks, and we denote the number of blocks in each stratum by d. Suppose we have d available workers, in order to iterate all regions of X, we need *d*^3^ blocks and thus *d*^2^ strata. For a stratum s we have d blocks $Z_{i}^{\left(s\right)}\;\text{for}\;i=0,1,\cdots ,n-1$. A detailed partition function for a size of *I* × *J* × *K* tensor X is provided below:

(4) $$\;b_{i}=\left(i\left\lceil I/d\right\rceil ,\left(i+1\right)\left\lceil I/d\right\rceil \right)\;\;\;\;b_{j}=\left(j\left\lceil J/d\right\rceil ,\left(j+1\right)\left\lceil J/d\right\rceil \right)\;b_{k}=\left(k\left\lceil K/d\right\rceil ,\left(k+1\right)\left\lceil K/d\right\rceil \right)\;\;\;\;\;\;\;\;j_{s,i}=\left(j+s\right)\;\;\;\;\;k_{s,i}=\left(j+s\right)\;\text{mod}\;d\;\;\;\;\;Z_{i}^{\left(s\right)}=X_{\left(b_{i},b_{j}{}_{{}_{s,i}},b_{k_{s,i}}\right)}$$

**Figure 2:**
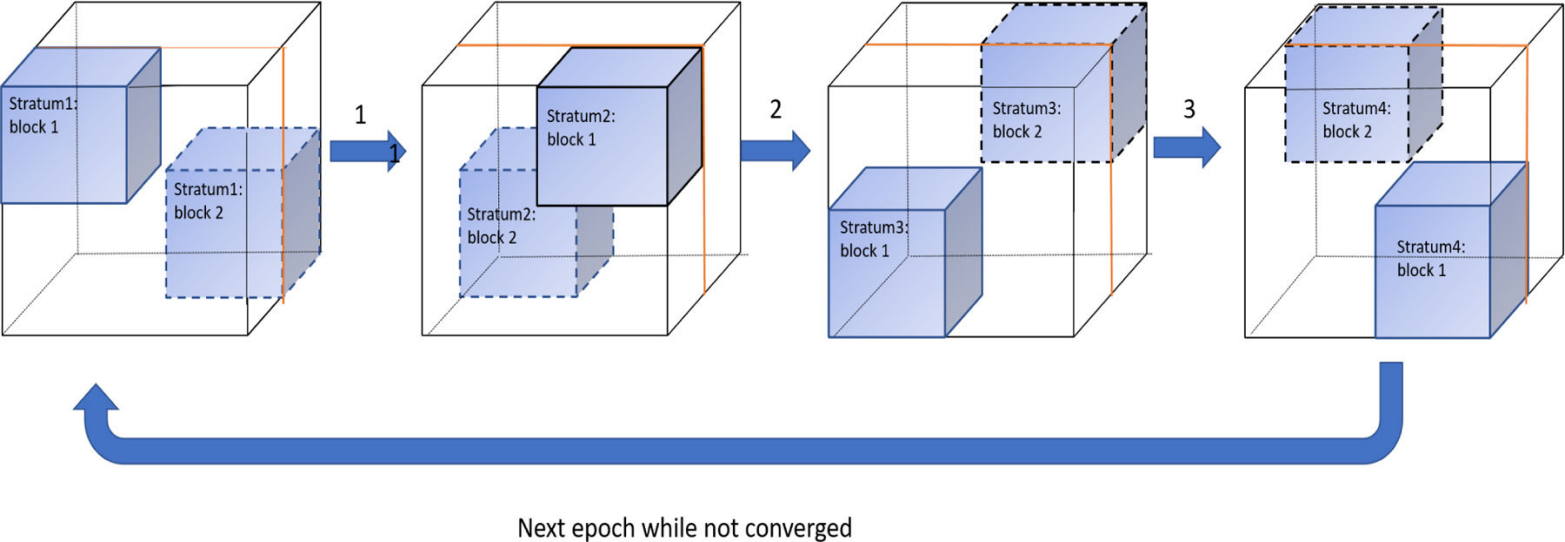
A graphical example of our SGranite: Suppose there are 2 workers, we will have 8 blocks and 4 strata after partition. We run this process iteratively until convergence. In each epoch, start from strata one, each worker runs SGD for its own assigned block in parallel. Check the convergence until all strata are iterated. We repeat above algorithm again if the stopping criteria is not satisfied. All intermediate results are saved as Resilient Distributed Datasets (RDD) collections and cached in memory.

Figure 2 provides an example of how to divide a count tensor for 2 available workers.

#### 3.4.2 Block Parallelization.

**Figure 3:**
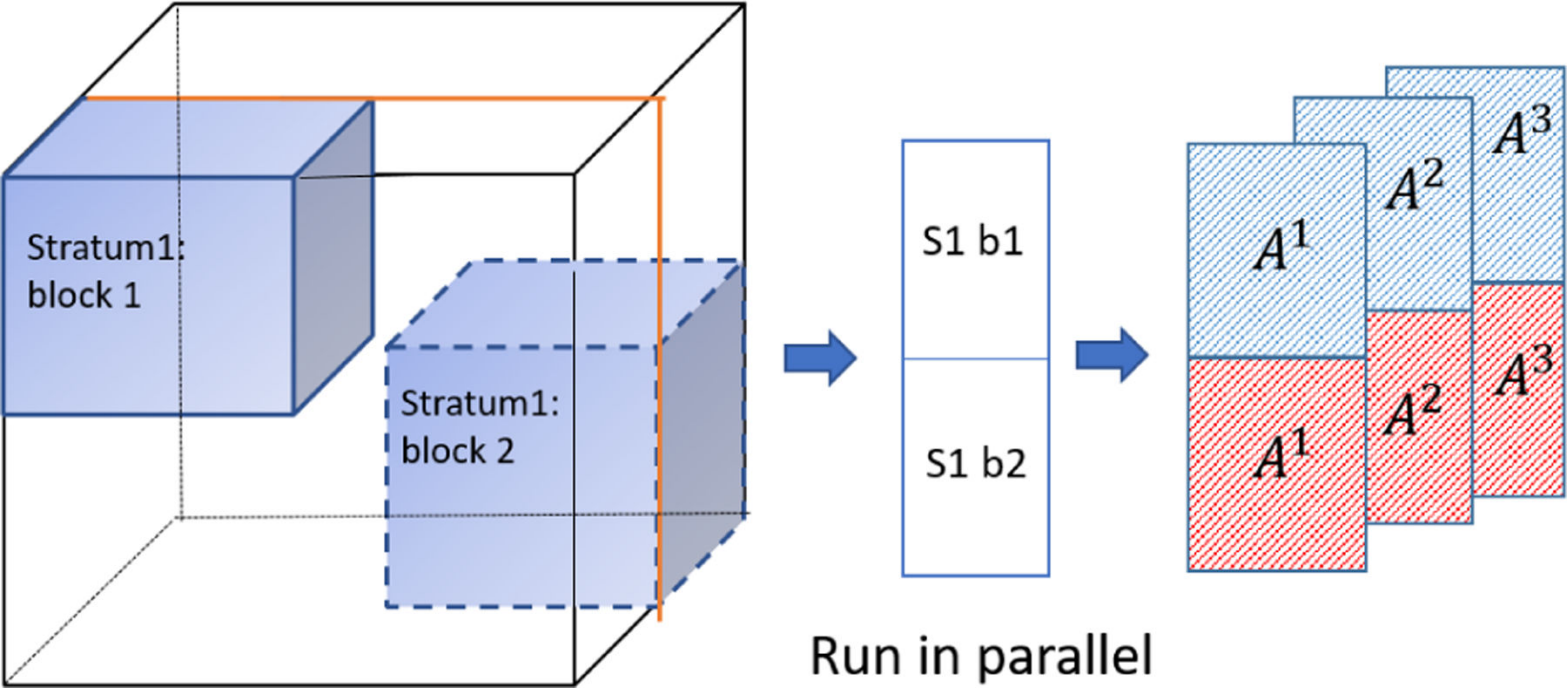
**A graphical example of one stratum training: Given one stratum of training data and factor matrices** *A*^(1)^, *A*^(2)^, *A*^(3)^, **we run SGD on each block in parallel. Then factor matrices** *A*^(1)^, *A*^(2)^, *A*^(3)^ **are updated and used as the initialization for the next stratum training.**

Prior to introducing how SGranite iteratively solves the optimization problem in parallel, we introduce some definitions. A full epoch is defined as when the algorithm has seen all the *d*^3^ blocks in the tensor. Since we need *d*^2^ strata to cover all the blocks, we need to perform *d*^2^ inner iterations. Therefore, we refer to each stratum training as a single inner iteration. Thus, in SGranite, the computation of each stratum is performed sequentially in each epoch. But for each stratum, we run SGD on the *d*^2^ blocks in parallel. After each inner iteration, we update the factor matrices and use them as the initialization for the next stratum. Figure 3 provides an example of training using a single stratum. Upon the completion of an epoch (all strata have been run), the factor matrices are combined from all the workers, and then renormalized for identifiability. The normalization can be performed for a user-specified mode, otherwise it defaults to the first mode. Convergence is checked between epochs by measuring the changes in the KL divergence to see if it is below a given tolerance. The details for the parallel-version of SGranite is described in Algorithm 2.

#### 3.4.3 Spark Implementation Details.

The non-zero elements of the count tensor are stored in a list using the coordinate format and loaded as Resilient Distributed Datasets (RDDs), and then it is shared throughout our cluster as a broadcast variable. A broadcast variable in Spark is immutable, meaning that it cannot be changed later on. This may seem inconvenient but it truly suits our case since we only need to read values from the tensor to calculate gradients in each iteration.

$$\begin{array}{l} \bar{\underline{\text{Algorithm}\;2\;\text{SGranite}\;\;\;\;\;\;\;\;\;\;\;\;\;\;\;\;\;\;}}\\ \;\;1:\;\;\text{Randomly initialize}\;〚{\lambda ;\text{A}}^{\left(\text{1}\right)}{;\cdot \cdot \cdot ;\text{A}}^{\left(N\right)}〛\\ \;\;2:\;\;\text{Partition the tensor and construct}\;d^{2}\;\text{stratas using}\;4\\ \;\;3:\;\;\text{for}\;m=1:M\;\text{do}\\ \;\;4:\;\;\;\;\;\;\text{for}\;l=1:d^{2}\;\text{do}\\ \;\;5:\;\;\;\;\;\;\;\;\;\;\;\text{Assign each block in}\;l\text{th strata to a worker}\\ \;\;6:\;\;\;\;\;\;\;\;\;\;\;\text{Each worker runs Algorithm 1 in parallel}\\ \;\;7:\;\;\;\;\;\;\;\;\;\;\;\text{Update the factors}\;〚{\lambda ;\text{A}}^{\left(\text{1}\right)}{;\cdot \cdot \cdot ;\text{A}}^{\left(N\right)}〛\\ \;\;8:\;\;\;\;\;\;\;\text{end for}\\ \;\;9:\;\;\;\;\;\;\text{Gather results from each worker}\\ \;\;10:\;\;\;\;\;\text{Normalize factor matrices according to the specified mode}\\ \;\;11:\;\;\;\;\;\text{if}\;\text{converged}\;\text{then}\\ \;\;12:\;\;\;\;\;\;\;\;\;\;\;\text{break}\\ \;\;13:\;\;\;\;\;\text{end if}\\ \;\;14:\;\;\text{end for}\\ \underline{\;\;15:\;\;\text{Return}\;〚{\lambda ;\text{A}}^{\left(\text{1}\right)}{;\cdot \cdot \cdot ;\text{A}}^{\left(N\right)}〛\;\;\;\;\;\;\;\;\;\;\;\;\;\;} \end{array}$$

We do not broadcast factor matrices since we need to update them in each iteration. Due to our partition function, each worker has a chance to update factors matrices with different boundaries. The best way is to partition factor matrices using Block ID. In this way, we can reduce the memory and communication cost. Specifically, we applied map and aggregateByKey functions to partition the factor matrices into blocks. The function map transforms each entry of the sparse tensor into an element in the RDD whose key is a block ID. Then aggregateByKey groups each block together and persists in memory. In each inner iteration, we use groupWith to build a stratum partitioned using partitionBy and then use mapPartitions to assign tasks to each node.

We found storing factor matrices RDDs and partitioned result in a significant acceleration, but not doing this will cause virtual memory issues in our experiments. Our experiments suggest such a design will enable us to obtain better speed-up and scalability.

## 4 EXPERIMENTS AND RESULTS

In this section, we first provide descriptions for two real-world health datasets. We then give an overview of baseline methods and provide qualitative and quantitative results.

### 4.1 Datasets

We use the following two publicly available datasets:

- Influenza: Using Google Flu Trends historical data^2^ from 2003 to 2015, we generated a tensor to uncover temporal influenza patterns that are unique and similar across multiple states. For each region in the United States, we collected the number of search queries related to influenza on a weekly basis over 11 years. The resulting tensor is 12 regions × 52 weeks × 11 years. Although the data quality has been shown to be low Olson, Donald R et al. [28], this dataset is used to demonstrate the feasibility of SGranite on search data.
- MIMIC-III [20]: MIMIC-III is large database containing deidentified health data associated with approximately sixty thousand admissions of critical care unit patients from the Beth Israel Deaconess Medical Center collected between 2001 and 2012. For each patient, we extract medications and the International Classification of Diseases (ICD-9) diagnosis codes. ICD-9 codes are aggregated using Clinical Classification Software (CCS) categories^3^, a standard preprocessing step in healthcare analysis. Similarly, medications are grouped using the Anatomical Therapeutic Chemical (ATC) Classification via the RxNorm RESTful Web API, a web service developed by the National Library of Medicine^4^. The aggregation step results in a 38159 patient × 234 diagnosis × 511 medication tensor.

### 4.2 Baseline Approaches

We will compare SGranite to both centralized and distributed CP decomposition methods.

- CP-APR [8]: The first algorithm proposed for modeling sparse count data using a Poisson distribution. There is no support for constraints, and the updates are performed using multiplicative updates. The algorithm has been ported to Python by the authors of [16].
- Granite [16]: A centralized extension of CP-APR that incorporates the angular penalty, *ℓ*_2_, and the simplex projection as regularization terms. The authors shared a python implementation of Granite fit using SGD.
- FlexiFact [6]: A distributed algorithm based on the DSGD approach that factorizes a coupled tensor and matrix using a similar partition method. However, it uses least squares as an objective and only supports non-negativity and *ℓ*_1_ constraints. For a fair comparison, we implemented the algorithm in Spark according to the paper.

### 4.3 Implementation and Hardware Details

SGranite is implemented in Python and the source code is publicly available^5^. The experiments were conducted on AWS. The cluster has one master and three worker nodes. Each node has four virtual cores and 16 GB of RAM. Results in our paper were reported using 4 workers.

#### 4.3.1 Hyperparameter tuning.

The logistic regularization penalty *β*_3_, simplex projection *β*_2_ and the angular penalty *β*_1_ were set as 0.06, 0, and 0.02, respectfully. *θ*_*n*_ for angular penalty for each mode, the learning rate, and batch sizes were selected as 0.9, 0.0001, and 200 respectively. All hyperparameters were chosen based on a grid search over values.

### 4.4 Results

#### 4.4.1 Scalability and Accuracy.

**Figure 4:**
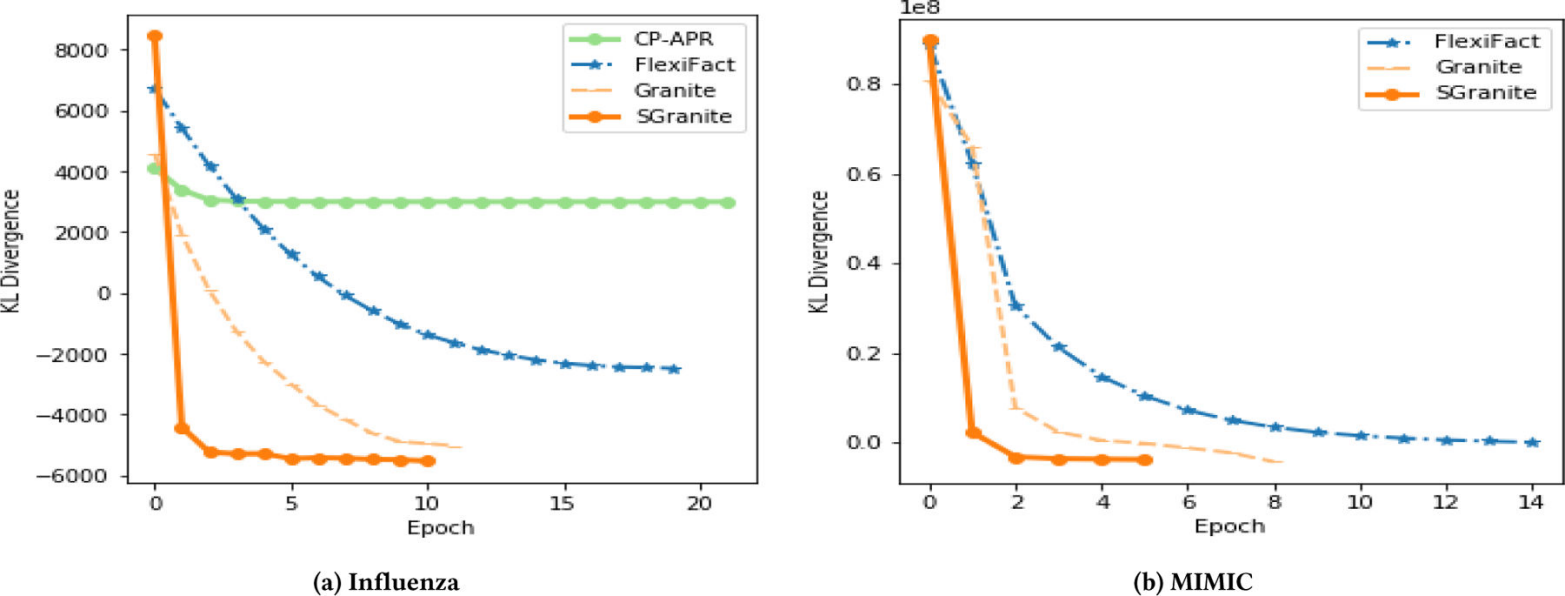
Comparison of two distributed and two non-distributed CP models using KL divergence. SGranite converges in less epochs than the other methods. The negative KL divergence arises from the fact that the observed values are not probability measurements.

First, we assess the quality of the approximation (measured by KL divergence) for SGranite and the other baseline methods. Figure 4 shows the KL divergence as the function of the number of epochs on both datasets. For the centralized algorithms (CP-APR and Granite), each epoch corresponds to a full iteration. The plots demonstrate that SGranite converges at least 4× faster than FlexiFact and also faster than the centralized algorithms. Moreover, the quality of the approximation is better than any of the existing methods. This suggests that SGD-based methods may help escape undesirable local minima (compared to CP-APR). The figure also highlights the importance of appropriately modeling the data distribution as opposed to using the least-squares loss (FlexiFact) may not yield the best approximation.

**Figure 5:**
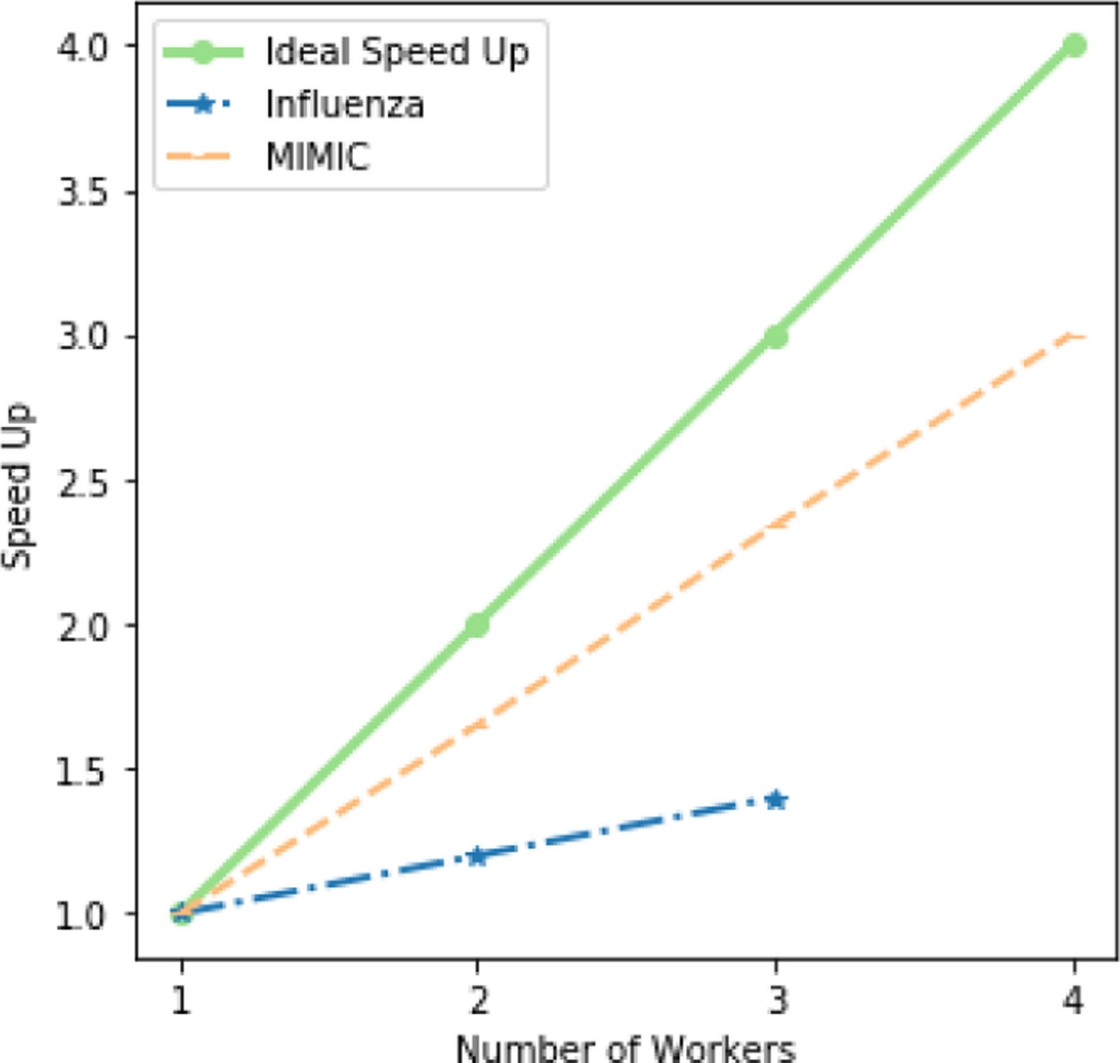
The speed-up curve for both datasets. It shows analysis of large datasets will gain an obvious speed up by using SGranite.

Next, we evaluate the scalability of our algorithm with respect to the number of workers. We calculate the speed-up as the ratio between the total execution time and the sequential execution time. Figure 5 demonstrates the speed-up of SGranite with respect to the number of workers. As can be seen in the figure, the speed up for the MIMIC tensor is very close to the ideal speed-up, as it is relatively large. However, there is a limited improvement on the Influenza tensor, a small dataset. This is due to the communication cost that is incurred in coordinating the different nodes. We note that because SGranite caches the updated factor matrices in memory to minimize disk accesses between consecutive iterations, it is able to scale to a large dataset and a large number of workers. Since this speed-up would not be possible on a system like Hadoop, we do not provide a comparison with FlexiFact.

#### 4.4.2 Qualitative and Quantitative Assessment of the Constraints.

**Figure 6:**
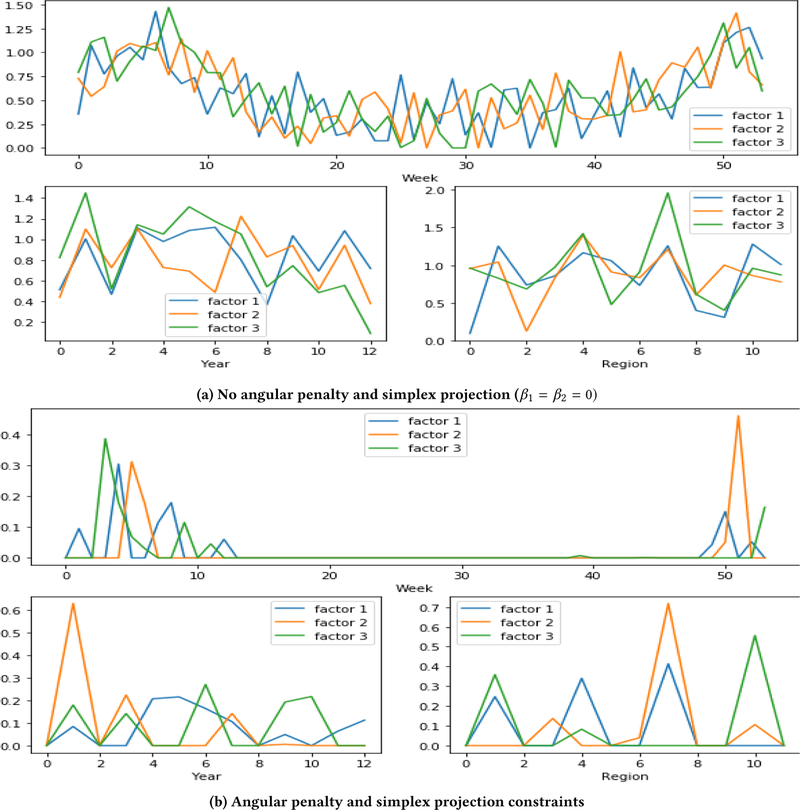
**A comparison of the learned latent factors with and without constraints using** *R* = 3. **Year from 2003 to 2015**

To examine the impact of the constraints, we first compare the results from SGranite on the influenza dataset both with and without the angular and simplex regularization terms. Figure 6a shows the latent factors learned without the regularization terms, and Figure 6b shows the latent factors learned with the regularization terms. From these plots, we observe that the learned factors without regularization are highly correlated and can be difficult to distinguish from one another. In Figure 6a, it is hard to discern any noticeable pattern across the weeks and the different regions. In comparison, Figure 6b demonstrates the potential of incorporating both diversity and sparsity. We can observe that factor 2 predominantly captures the peak influenza season that occurs both towards the end of December and in mid-February in region 7, whereas factor 3 is slightly delayed and captures the influenza trend in regions 1 and 10. Furthermore, all three factors capture the peak in influenza season that occurs in late December and early February through March.

Next, we quantitatively assessed the impact of the logistic regression and angular penalty on the MIMIC-III dataset. To evaluate the discriminative power and distinctiveness of the learned factors, we used the in-hospital mortality cohort similar to that proposed in [21]. We used 37,000 patients, including all 5,014 patients who died during admission. We split our dataset into 80% training and 20% testing. We measured the discrimination on the test set using the area under the receiver operating characteristic curve (AUC). Distinctiveness is measured using the average overlap or the degree of overlapping between latent factors. It is defined as the average of cosine similarities between all latent factor pairs:

$$\text{Avg}\;\text{Overlap}\;\text{=}\;\frac{\sum_{r_{1}}^{R}\sum_{r_{2}>r_{1}}^{R}\text{cos}(a_{r_{1}}^{\left(2\right)},a_{r_{2}}^{\left(2\right)})+\text{cos}(a_{r_{1}}^{\left(3\right)},a_{r_{2}}^{\left(3\right)})}{R\left(R-1\right)}$$

Table 3 summarizes the AUC, total computation time (or running time), and the average overlap. We observe that SGranite can not only accelerate the tensor decomposition but also provides better prediction than other baseline methods. Moreover, the average over-lap is smaller than Granite even without the angular constraints. This suggests that the partition function may also have some beneficial impact in terms of reducing overlapping factors. Moreover, incorporating the angular constraints further helps the discriminative ability of the model. This suggests that adding diversity constraints to yield less correlated latent features may also help the resulting predictive model. Therefore, SGranite supports a variety of flexible constraints and yields improved predictive performance.

#### 4.4.3 Case Study 1: Flu Patterns.

We provide a further qualitative assessment of our learned latent patterns from the influenza dataset. First, we comment on the ability to capture the overall flu season trends. Although flu season can vary across region to region, the flu season is typically between October through May (week 43 to week 22) [1]. We observe this phenomenon even with and without angular penalty constraints as illustrated in Figure 6. The variance in region and slight shifts in the week are further evident when angular penalty and simplex projection constraints are present (Figure 6b). We can see that some of the regions are present only in 1 of the factors. Moreover, slight shifts along the week are observed (top chart), depending on which latent factor with the higher elements occurring between weeks 48 and 13. This provides further confirmation that each region will have slightly different times when influenza will be more prominent.

**Figure 7:**
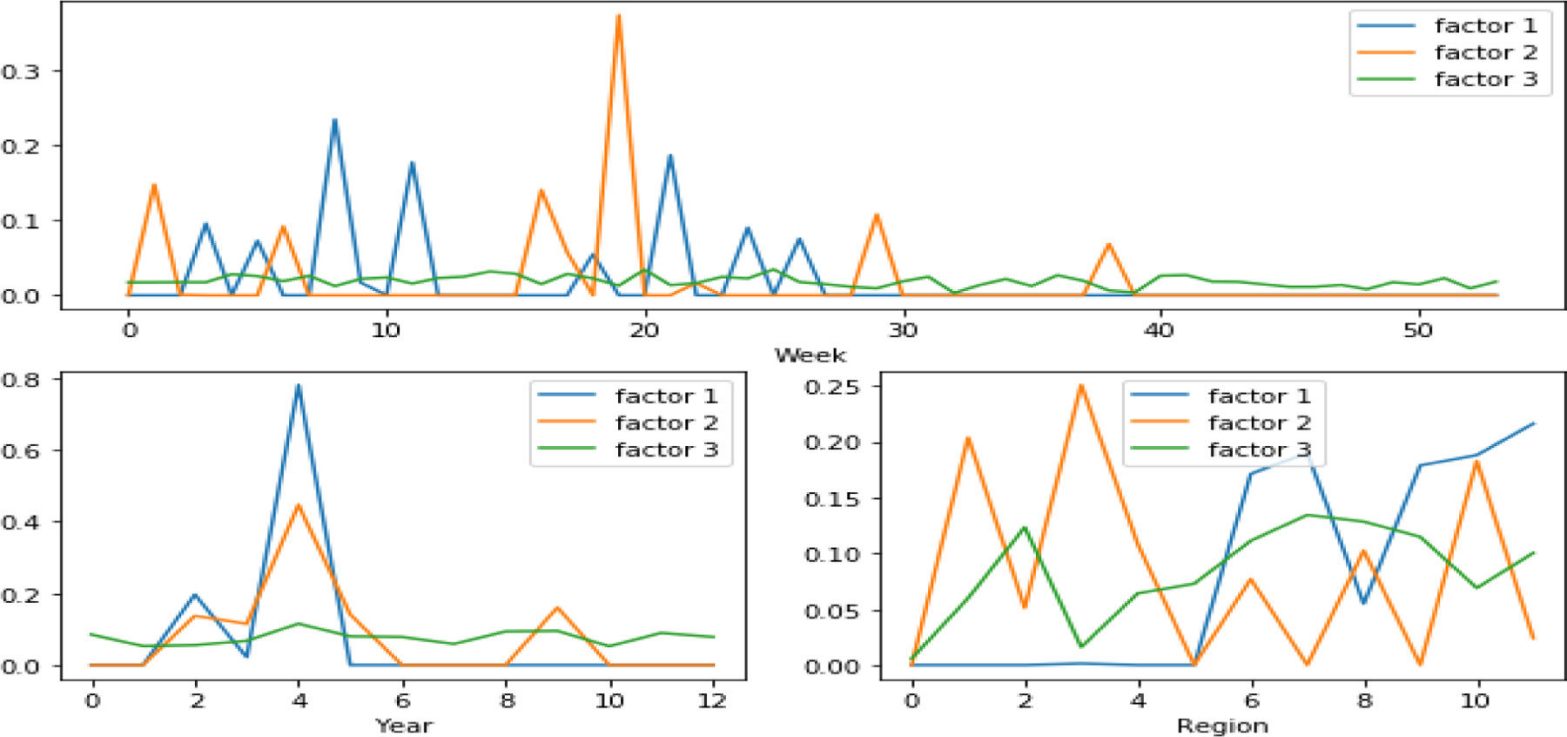
Latent factors obtained using Flexifact. Year from 2003 to 2015

**Figure 8:**
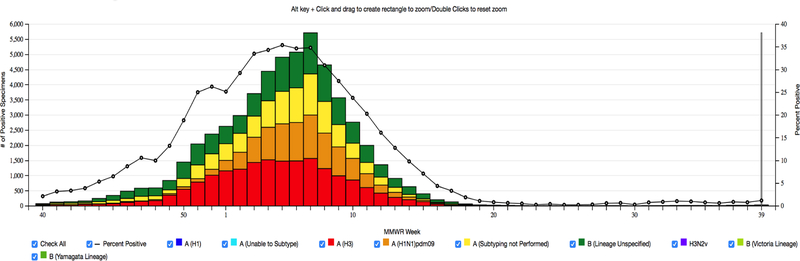
This figure is downloaded from CDC, it shows the actual influenza positive tests reported to CDC in 2010–2011, week ending Oct 01, 2001.

We also assessed the learned flu patterns with FlexiFact, the other distributed CP algorithm that supports non-negativity and sparsity. Figure 7 presents the learned latent factors using FlexiFact. We observe that the peak level regions that are discovered using SGranite are more consistent with the CDC influenza positive test results, shown in Figure 8. The peaks that are discovered by FlexiFact are inconsistent with the observed CDC reports. FlexiFact factors suggest two different peaks, one between weeks 8–10 and one 18–20, whereas the CDC reports note a peak around 7–9 and by week 20, it has mostly died down. Moreover, we observe that the FlexiFact latent factors are more difficult to interpret as the region and year factors are fairly correlated. We also compared with the learned factors from a previous study [13] and found our learned patterns were more consistent with the observed results. This suggests that the incorporation of constraints not only improves interpretability but also provides robustness to noise.

#### 4.4.4 Case Study 2: Phenotypes.

We conducted a second case study to examine SGranite’s ability to extract discriminative and distinct clinical characteristics from the MIMIC III dataset. The identification of clinical phenotypes from EHR data can help advance our understanding of disease risk and drug response as well as support the practice of precision medicine on a national scale [33, 38].

For clinicians, diversity is important to discover rare phenotypes in a patient population. Moreover, diverse phenotypes are likely easier to implement, as a clinician may find it difficult to rank-order or apply phenotypes that have substantial overlap. In addition, discriminative phenotypes are better predictors of mortality (shown in Table 3) and thus can be used to assist the decision-making process.

Table 4 presents the learned phenotypes that are important where importance is determined based on the magnitude of the phenotypes (or λ*r* ). Thus, these are the three sets of patient characteristics at which diagnosis and medication are dominant. First, we observe that the learned phenotypes have limited number of overlapping elements. In table 4, the most significant phenotype (λ_1_ ) captures acute complications with heart diseases which can be riskier. In particular, acute respiratory distress syndrome has a mortality rate of 30–50% and is associated with long hospital stays [27]. The third phenotype (λ_3_) captures more chronic diseases such as heart valve disorder, leukemias, and osteoarthritis. In addition, we observe that most medication codes in Table 4 are associated with diagnosis codes above. For example, potassiuman chloride and practolol are commonly used to lower blood pressure in hypertensives [10, 37]. An ACE inhibitor is used primarily for the treatment of hypertension and congestive heart failure [26]. And magnesium carbonate has shown to be effective for chronic kidney diseases and intracranial injury [5, 35].

## 5 CONCLUSION

In this paper, we presented a distributed, diverse, non-negative tensor decomposition framework that supports a variety of constraints including an angular penalty to encourage diversity and a simplex projection to encourage sparsity while scaling to large tensors. By imposing such regularization terms, SGranite successfully extracts meaningful latent factors in two real-world use cases. Moreover, by using Spark, SGranite successfully reduces processing time by dramatically reducing the workload and high communication cost. In addition, SGranite improves binary prediction tasks by incorporating logistic supervision into the fitting process. In the future, we plan to develop a distributed algorithm that can handle linear regression problem and also an extension that can use outside data sources as the guidance information.

## Figures and Tables

**Table 1:** A comparison of the supported features between SGranite versus state of arts methods

Feature	SGranite	CP-APR [8]	Granite [16] (fit using SGD)	FlexiFact[6]	DisTenC [11]
Scalable	Yes	No	Yes	Yes	Yes
Memory Efficient	Yes	No	Yes	No	Yes
Time efficient	Yes	No	No	Yes	Yes
Appropriate for count data	Yes	Yes	Yes	No	No
Works with constraints	Yes	No	Yes	Yes	No

**Table 2:** Table of symbols and their associated definitions

Symbol	Definition
X, **X**, **x**, x	Tensor, Matrix, Column Vector, Scalar
**X**_(*n*)_	*n*-mode matricization of a tensor X
**X**(**r**, :)	*r*th row of **X**
**X**(:, **r**)	*r*th column of **X**
**A**^(*n*)^	*n*th factor matrix
‖*a*‖_2_, ‖**A**‖_*F*_	*l*_2_ norm, Frobenius norm
*	Hadamard (elementwise) product
○	outer product
⊗	Kronecker product
⊙	Khatri-Rao product (column-wise ⊗)

**Table 3:** Table of AUC, running time, and average overlapping using different methods. The highest AUC value means extracted phenotypes have stronger discrimination. The lowest running time indicates our distributed method can significantly accelerate the computation time. Compared to CP-APR and FlexiFact, adding angular penalty improved the distinction significantly.

Model	AUC	Time	Avg Overlap
CP-APR [8]	0.63	> 1 hour	0.3
FlexiFact [6]	0.65	35 mins	0.37
Granite [16]	0.67	> 1 hour	0.3
SGranite (*β*_1_ = 0)	0.68	**20 mins**	0.1
SGranite (*β*_1_ > 0)	**0.71**	25 mins	**0.07**

**Table 4:** Table of top 3 phenotypes with high *λ*. Upper four rows are diagnosis codes and four rows below are medication codes correspondingly

Phenotype 1: *λ*_1_ = 87	Phenotype 2: *λ*_1_ = 79	Phenotype 3: *λ*_3_ = 77
Respiratory distress syndrome	Coronary atherosclerosis & other heart disease	Heart valve disorders
Acute cerebrovascular disease	Intracranial injury	Intracranial injury
Coronary atherosclerosis & other heart disease	Congestive heart failure; nonhypertensive	Leukemias
Hypertension w/ complications & secondary hypertension	Acute and unspecified renal	Osteoarthritis

Potassium chloride	Oxyphenisatine	Practolol
Sodium chloride	Adrenergics, inhalants	Magnesium carbonate
Omeprazole	ACE inhibitors	Anilides
Potassium chloride	Nitroprusside	Sodium chloride

## CCS CONCEPTS

• Information systems → Web mining; Data extraction and integration; • Computing methodologies → Factor analysis; Canonical correlation analysis; MapReduce algorithms; • Applied computing → Health informatics

## Footnotes

1 https://github.com/hehuannb/SGranite-WWW

2 https://www.google.org/flutrends

3 The mapping from ICD-9 to CCS can be found at https://www.hcup-us.ahrq.gov/toolssoftware/ccs/ccs.jsp.

4 Details for the RxNorm RESTful Web API can be found at https://mor.nlm.nih.gov/download/rxnav/RxNormAPIREST.html.

5 https://github.com/hehuannb/SGranite-WWW
